# Gold Nanoclusters Synthesized within Single-Chain Nanoparticles as Catalytic Nanoreactors in Water

**DOI:** 10.3390/polym16030378

**Published:** 2024-01-30

**Authors:** Jokin Pinacho-Olaciregui, Ester Verde-Sesto, Daniel Taton, José A. Pomposo

**Affiliations:** 1 Centro de Física de Materiales (CSIC–UPV/EHU)–Materials Physics Center MPC, Pº Manuel Lardizabal 5, E-20018 Donostia, Spain; jokin.pinacho@ehu.eus (J.P.-O.);; 2 Laboratoire de Chimie des Polymères Organiques (LCPO), Université de Bordeaux INP–ENSCBP, 16 av. Pey Berland, 33607 Pessac CEDEX, France; 3 IKERBASQUE–Basque Foundation for Science, Plaza Euskadi 5, E-48009 Bilbao, Spain; 4 Departamento de Polímeros y Materiales Avanzados, Física, Química y Tecnología, University of the Basque Country (UPV/EHU), Pº Manuel Lardizabal 3, E-20800 Donostia, Spain

**Keywords:** single-chain nanoparticles, gold nanoclusters, catalytic nanoreactors

## Abstract

Metalloenzymes are able to catalyze complex biochemical reactions in cellular (aqueous) media with high efficiency. In recent years, a variety of metal-containing single-chain nanoparticles (SCNPs) have been synthesized as simplified metalloenzyme-mimetic nano-objects. However, most of the metal-containing SCNPs reported so far contained complexed metal ions but not metal nanoclusters (NCs) with diameter <5 nm, which could be used as powerful, emerging catalysts. Herein, we report the synthesis of gold nanoclusters (Au-NCs) within SCNPs and the further use of Au-NCs/SCNPs as catalytic nanoreactors in water. We demonstrate that a common motif contained in several drugs (i.e., the aminophenyl–oxazolidinone fragment present in Rivaroxaban, Sutezolid, and Linezolid) can be efficiently prepared in water from a hydrophobic precursor compound by using the Au-NCs/SCNPs as efficient catalytic nanoreactors. In summary, this work paves the way forthe synthesis of metal–NCs/SCNPs for advanced catalysis in aqueous media.

## 1. Introduction

Metalloenzymes contain metal ions as co-factors for a variety of biochemical reactions that take place in the cellular environment [1]. The precise outer coordination sphere offered by the metalloenzyme is critical for its catalytic activity and substrate selectivity. Hence, metalloenzyme activity is substantially reduced when the polypeptide 3D structure is altered due to, e.g., a severe pH change or heating. Consequently, many attempts have been performed to endow synthetic polymers with metalloenzyme-mimetic activity, trying to capitalize on the stability of polymers and the efficient catalytic activity of metal ions as co-factors [2,3]. Moreover, the folding of discrete synthetic polymer chains to single-chain nanoparticles (SCNPs) has introduced the additional advantage of the presence of local pockets within which metal ions could find an appropriate coordination sphere for efficient catalysis [4,5,6]. Several recent reviews disclose the potential advantage of SCNPs as metalloenzyme-mimetic catalysts [7,8,9,10,11].

However, most of the metal-containing SCNPs reported so far contained complexed metal ions but not entrapped metal nanoclusters (NCs) with diameter < 5 nm, which could be used as powerful, emerging catalysts. Interestingly, a pioneering work by Zhao et al. [12] reported the kinetics of the formation of gold nanoparticles (Au-NPs) using SCNPs decorated with tertiary amines without the involvement of any other additional reductants. In a subsequent work, the CO_2_-responsiveness of these SCNPs was used to produce gas-tunable nanoreactors for Au-NPs synthesis [13]. However, even if the localized surface plasmon resonance (LSRP) band characteristic of Au-NPs was observed by ultraviolet–visible (UV-Vis) spectroscopy, these authors did not investigate the catalytic properties of the resulting Au-NPs/SCNPs.

Metal nanoclusters offer unique advantages over complexed metal ions, including their small size, enhanced stability, and tunable properties for a wide range of applications in fields such as catalysis, biomedicine, energy, and electronics. In this work, we report the synthesis of gold nanoclusters (Au-NCs) with diameter < 5 nm within SCNPs and the further use of Au-NCs/SCNPs as catalytic nanoreactors in water. Gold nanoclusters have recently attracted great interest due to their small size and atomically precise number of atoms per nanocluster that offer interesting possibilities for a range of applications, including cancer phototherapy [14], bioimaging [15],sensing [16], andcatalysis [17]. Ligand-protected Au-NCs with a size between 2 and 5 nm are often used in catalysis [18]. The first use of Au-NCs for catalysis was reported by Haruta et al. [19] for the oxidation of CO even at very low temperatures (−70 °C). Since then, the catalytic properties of naked and ligand-protected Au-NCs have been investigated in a wide number of chemical transformations [20,21,22,23]. When compared to Au-NPs, the larger surface-to-volume ratio of Au-NCs explains their enhanced reactivity and superior catalytic properties. Moreover, the chemical inertness of gold nanoclusters compared to other metallic nanoclusters makes them highly stable in various environments and ensures long-term performance and durability in catalysis [20,21,22,23]. However, to the best of our knowledge, Au-NCs have not been synthesized within SCNPs, nor have the resulting SCNP-protected Au-NCs been employed as catalytic nanoreactors yet. Herein, we use an amphiphilic random copolymer self-assembled as SCNPs featuring beta-ketoester units as a template to generate and stabilize Au-NCs with diameter < 5 nm. The precise morphology of Au-NCs/SCNPs was revealed by transmission electron microscopy (TEM), whereas the hydrodynamic size in water was determined by dynamic light scattering (DLS) measurements. As proof-of-concept experiments of the catalytic activity of Au-NCs/SCNPs in water, we illustrate the fast reduction of nitrophenol to aminophenol as well as the conversion of nitrobenzene to aniline with anexcellent yield. Finally, we carry out the efficient transformation in water of 3-(4-nitrophenyl)-1,3-oxazolidin-2-one to 3-(4-aminophenyl)-1,3-oxazolidin-2-one—a common motif contained in several drugs like Rivaroxaban, Sutezolid, and Linezolid—using the Au-NCs/SCNPs as catalytic nanoreactors.

## 2. Materials and Methods

### 2.1. Materials

Oligo(ethylene glycol) methyl ether methacrylate (OEGMA) (average *M*_n_∼ 300 Da), (2-acetoacetoxy)ethyl methacrylate (AEMA) (95%), 2,2-azobis(2-methylpropionitrile) (AIBN) (≥98%), triethylamine (Et_3_N) (>99%), 1,4-dioxane (anhydrous, 99.8%), *n*-hexane (anhydrous, 95%), ethyl acetate (anhydrous, 99.8%), deuterated dimethyl sulphoxide (DMSO-d_6_) (99.9 atom % D), 4-cyano-4-(thiobenzoylthio)pentanoic acid (≥97%), 4-nitrophenol (≥99%), nitrobenzene (≥99%), 3-(4-nitrophenyl)-1,3-oxazolidin-2-one (95%), chloroform (CHCl_3_) (≥99%), and deuterated chloroform (CDCl_3_) (99.8 atom % D) were supplied by Merk (Sigma-Aldrich) (Madrid, Spain). Tetrachloroauric (III) acid trihydrate (99%) was supplied by Thermo Scientific (Eindhoven, The Netherlands). Methanol (MeOH, analytical grade) and tetrahydrofuran (THF, GPC grade) were supplied by Scharlau (Barcelona, Spain). Deionized water was obtained from a Thermo Scientific apparatus (Barnstead TII Pure Water System) (Eindhoven, The Netherlands).

### 2.2. Techniques

Size exclusion chromatography (SEC) measurements were conducted at a temperature of 30 °C in an Agilent 1200 system that was equipped with PLgel 5 μm Guard and PLgel 5 μm MIXED-C columns. The measurements employed a triple detection system, which included a differential refractive index detector (Optilab Rex, Wyatt, Toulouse, France), a MALLS detector (MiniDawn Treos, Wyatt, Toulouse, France), and a viscosimetric (VIS) detector (ViscoStar-II, Wyatt, Toulouse, France). The data obtained were analyzed using Wyatt’s ASTRA Software (version 6.1). THF was used as eluent with a flow rate of 1 mL/min. For both poly(OEGMA-co-AEMA) and the SCNPs, a value of dn/dc = 0.115 was used. ^1^H Nuclear Magnetic Resonance (NMR) spectra were obtained at room temperature (r.t.) in a Bruker (Madrid, Spain) spectrometer operating at 400 MHz using CDCl_3_ as solvent. Infrared (IR) spectra were recorded at r.t. on a JASCO 3600 (Madrid, Spain) FTIR spectrometer. Dynamic light scattering (DLS) measurements were carried out at r.t. on a Malvern Zetasizer Nano ZS (Cambridge, UK) apparatus. Ultraviolet–visible (UV-Vis) spectra were recorded in an Agilent 8453A (Madrid, Spain) UV-Vis spectrometer with a Peltier thermostatic cell holder T-controller 89096A. Transmission electron microscopy (TEM) experiments were conducted in a TECNAI G220 TWIN (Eindhoven, The Netherlands) high-resolution transmission electron microscope. The measurements were carried out with a 200 kV accelerating voltage, employing low dose conditions.

### 2.3. Procedures

#### 2.3.1. Procedure for the Synthesis of Poly(OEGMA-co-AEMA)

OEGMA (1.54 mL, 5.6 mmol), AEMA (0.26 mL, 1.38 mmol), 4-cyano-4-(thiobenzoylthio)pentanoic acid (18.3 mg, 0.065 mmol), and AIBN (2.15 mg, 0.013 mmol) were dissolved in 1,4-dioxane (3 mL), and the resulting mixture was degassed with argon for 15 min. The copolymerization reaction took place at 70 °C for 24 h, yielding poly(OEGMA-co-AEMA) as a pink oil product that was isolated by precipitation in hexane. Subsequently, the copolymer was redissolved in a minimal amount of THF and added to an excess of hexane (twice), followed by solvent removal and further drying at r.t. under vacuum. The content of AEMA units in poly(OEGMA-co-AEMA) was 35 mol%, as determined by ^1^H NMR spectroscopy.

#### 2.3.2. Procedure for the Synthesis of Gold Nanoclusters (Au-NCs) within Poly(OEGMA-co-AEMA) Single-Chain Nanoparticles (SCNPs)

In a typical reaction, poly(OEGMA-co-AEMA) (15 mg, 0.03 mmol) and tetrachloroauric (III) acid trihydrate (0.00245 mmol) were dissolved in water (15 mL) under argon at r.t. for 1 h. The reaction was followed by DLS, confirming that there was no significant change in hydrodynamic size of the poly(OEGMA-co-AEMA) SCNPs under such conditions. TEM measurements confirmed the presence of Au-NCs within the SCNPs. Additionally, UV-Vis measurements showed that the Au-NCs synthesized within the SCNPs did not have the localized surface plasmon resonance (LSPR) absorbance signal characteristic of gold nanoparticles (Au-NPs).

#### 2.3.3. Procedure for the Reduction of 4-Nitrophenol Catalyzed by Au-NCs/SCNPs

The Au-NC/SCNPs were used as catalyst for the reduction at r.t. of 4-nitrophenol (0.2 mmol) to 4-aminophenol in the presence of NaBH_4_ (0.8 mmol). In a typical experiment, a solution of 2 mL of Au-NC/SCNPs aqueous solution (1 mg/mL) was prepared and 18.8 µL of 4-nitrophenol and 30.35 mg of NaBH_4_ were sequentially added under argon into a vial at 0 °C. The 4-nitrophenol reduction reaction was then monitored via UV-Vis spectrophotometry. Absorbance was recorded by taking 1 µL of crude at a given reaction time, which was then diluted in 2 mL of deionized water. After reaction, 4-aminophenol was purified via preparative thin-layer chromatography (TLC) (*n*-hexane/ethyl acetate 1:1), with a yield of 95%. ^1^H NMR (400 MHz, DMSO-d_6_, ppm): δ 4.36 (s, 2H), 6.42–6.46 (m, 4H), 8.34 (s, 1H).

#### 2.3.4. Procedure for the Reduction of Nitrobenzene Catalyzed by Au-NCs/SCNPs

The Au-NC/SCNPs were used as catalysts for the reduction at r.t. of nitrobenzene (0.2 mmol) to aniline in the presence of NaBH_4_ (0.8 mmol). The same procedure described in Section 2.3.3 was followed. Absorbance was recorded by taking 2 µL of crude at a given reaction time, which was then diluted in 4 mL of deionized water. After reaction, aniline was purified via preparative TLC (*n*-hexane/ethyl acetate 1:1), with a yield of 96%. ^1^H NMR (400 MHz, DMSO-d_6_, ppm): δ 3.65 (s, 2H), 6.67–6.70 (m, 2H), 6.73–6.77 (m, 1H), 7.13–7.17 (m, 2H).

#### 2.3.5. Procedure for the Reduction of 3-(4-Nitrophenyl)-1,3-oxazolidin-2-one Catalyzed by Au-NCs/SCNPs

The Au-NC/SCNPs were used as catalysts for the reduction at r.t. of 3-(4-nitrophenyl)-1,3-oxazolidin-2-one (0.2 mmol) to 3-(4-aminophenyl)-1,3-oxazolidin-2-one in the presence of NaBH_4_ (0.8 mmol). The same procedure described in Section 2.3.3 was followed. Absorbance was recorded by taking 2 µL of crude at a given reaction time, which was then diluted in 10 mL of deionized water. It is worth noting that due to the heterogenous character of the reaction and to the vigorous generation of molecular hydrogen gas, aliquots for analysis were prepared ensuring only the aqueous phase was taken. The product, 3-(4-aminophenyl)oxazolidin-2-one, was purified via preparative TCL (*n*-hexane/ethyl acetate 1:1), with a yield of 89%. ^1^H NMR (400 MHz, CDCl_3_), ppm): δ 4.63 (s, 2H), 3.97–4.01 (dd, 2H), 4.43–4.47 (dd, 2H), 6.68–6.71 (d, 2H), 7.27–7.30 (d, 2H).

## 3. Results and Discussion

### 3.1. Synthesis of Gold Nanoclusters within Single-Chain Nanoparticles (Au-NCs/SCNPs)

**Figure 1:**
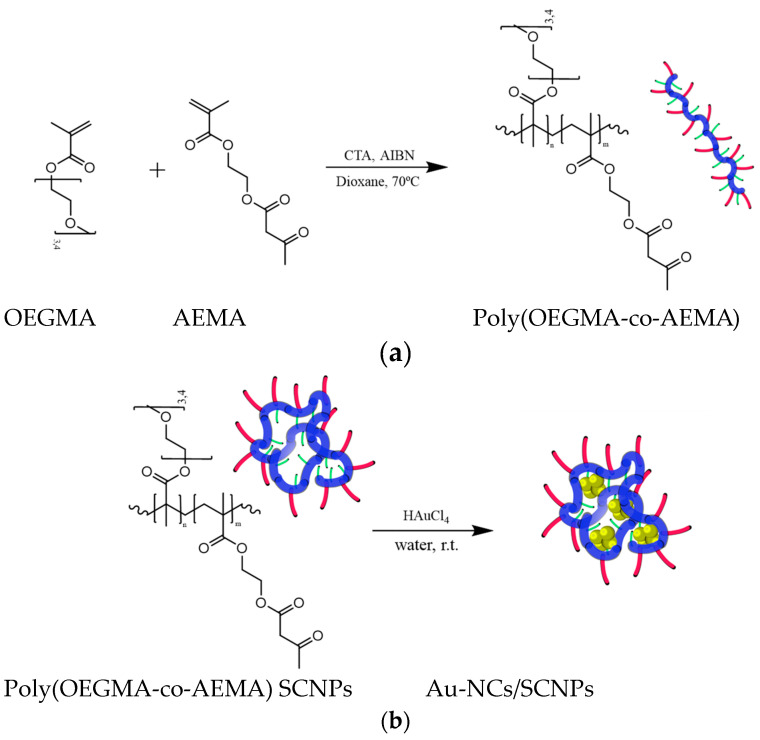
(**a**) Synthesis of an amphiphilic poly(OEGMA-co-AEMA) random copolymer featuring hydrophilic oligo(ethyleneglycol) methyl ether methacrylate (OEGMA) and hydrophobic (2-acetoacetoxy)ethyl methacrylate (AEMA) repeat units via reversible addition fragmentation chain-transfer (RAFT) polymerization. (**b**) Schematic illustration of the synthesis of Au-NCs/SCNPs from poly(OEGMA-co-AEMA) self-assembled in water in the form of SCNPs.

We targeted an amphiphilic poly(OEGMA-co-AEMA) random copolymer featuring hydrophilic oligo(ethyleneglycol) methyl ether methacrylate (OEGMA) and hydrophobic (2-acetoacetoxy)ethyl methacrylate (AEMA) units as a template for the synthesis of Au-NCs. Based on the literature data of low-molecular-weight ligands [24], we surmised that the beta-ketoester group of AEMA could be used as a reductant of Au(III) ions, as well as a stabilizing and structure-directing agent, to generate the Au-NCs. Poly(OEGMA-co-AEMA) was synthesized by means of reversible addition fragmentation chain-transfer (RAFT) polymerization (see Figure 1a).

**Figure 2:**
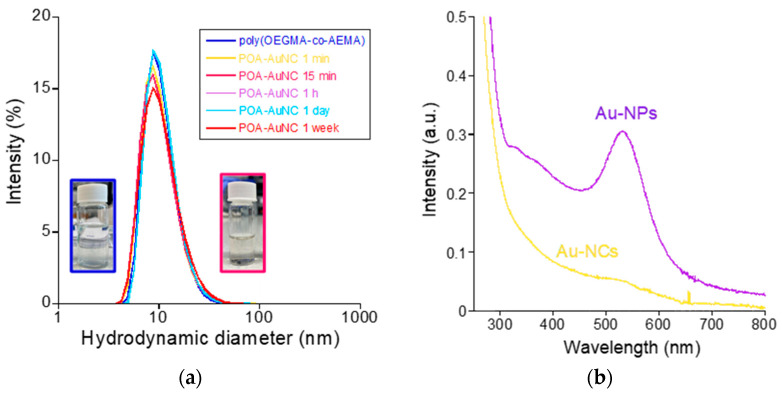
(**a**) DLS size distribution of poly(OEGMA-co-AEMA) SCNPs and Au-NCs/SCNPs. (**b**) Comparison of the UV-Vis spectra of Au-NPs synthesized in the presence of poly(OEGMA-co-AEMA) SCNPs at a (Au(III))/(beta-ketoester (AEMA)) ratio of 1 vs. Au-NCs synthesized within poly(OEGMA-co-AEMA) SCNPs at a (Au(III))/(beta-ketoester (AEMA)) ratio of 0.08 (see text).

The copolymer showed a weight-average molecular weight (*M*_w_) of 80.7 kDa and a low dispersity (*Ɖ*) value of 1.12, as determined by SEC. The content of AEMA units in the copolymer was 35 mol%, as estimated from ^1^H NMR spectroscopy. It is well-known from previous works that amphiphilic poly(OEGMA-co-AEMA) random copolymers with this AEMA content are able to self-assemble intramolecularly in water at high dilution (1 mg/mL) into discrete core–shell-like SCNPs [25,26]. In agreement with previous results, DLS measurements of poly(OEGMA-co-AEMA) in water at 1 mg/mL revealed an average hydrodynamic diameter of *D*_h_ = 11.0 nm corresponding to discrete, individual SCNPs without any sign of the presence of multi-chain aggregates (see Table 1 and Figure 2a).

**Figure 3:**
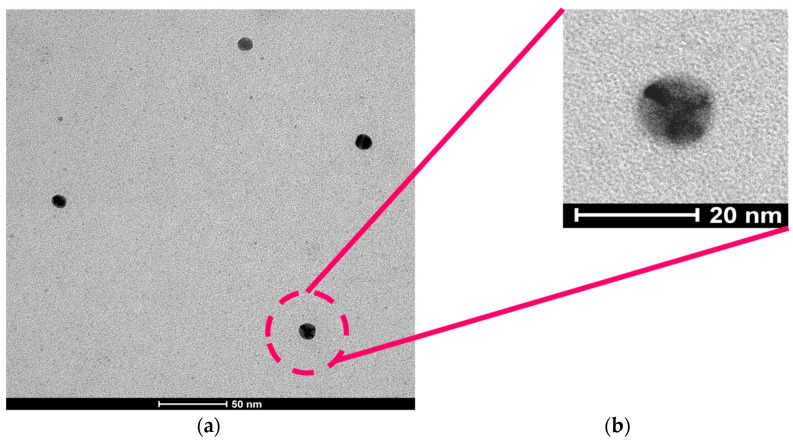
(**a**) TEM picture of Au-NCs synthesized within poly(OEGMA-co-AEMA) SCNPs at a (Au(III))/(beta-ketoester (AEMA)) ratio of 0.08. (**b**) Illustration of the presence of (darker) Au-NCs with a diameter < 5 nm within a representative single-chain nanoparticle of ca. 11 nm in diameter.

**Figure 4:**
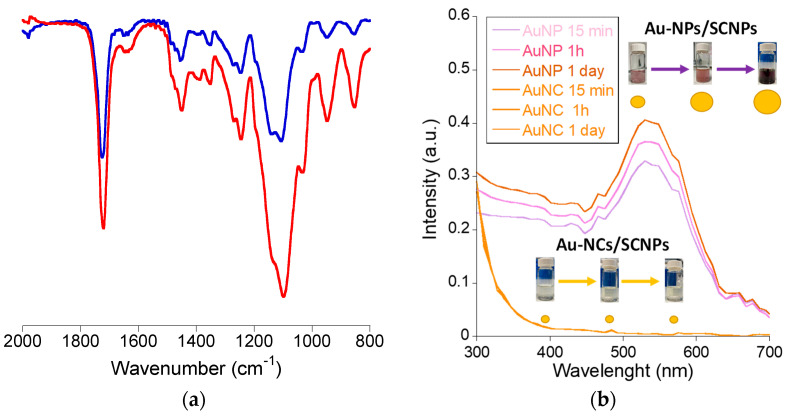
(**a**) IR spectra of neat poly(OEGMA-co-AEMA) SCNPs (blue color) and Au-NCs/SCNPs (red color). (**b**) Au-NPs synthesized in the presence of poly(OEGMA-co-AEMA) SCNPs at a (Au(III))/(beta-ketoester (AEMA)) ratio of 1 vs. Au-NCs synthesized at a ratio of 0.08.

For the synthesis of Au-NCs within the SCNPs through beta-ketoester-mediated Au(III) reduction (Figure 1b), we found control of the (Au(III)/(beta-ketoester (AEMA))ratio to be critical.Hence, by using a (au(III))/(beta-ketoester (AEMA)) ratio of 1, we obtained gold nanoparticles (Au-NPs) instead of Au-NCs, as revealed by the intense localized surface plasmon resonance (LSPR) UV-Vis absorbance signal characteristic of Au-NPs (Figure 2b). Interestingly, by lowering the (au(III))/(beta-ketoester (AEMA)) ratio to 0.08, we observed via TEM the presence of Au-NCs (diameter < 5 nm) within individual poly(OEGMA-co-AEMA) SCNPs (Figure 3), as well as the total absence of the LSPR band typical of larger Au-NPs (Figure 2b). No significant differences were found between the IR spectra of neat poly(OEGMA-co-AEMA) and the Au-NCs/SCNPs, as illustrated in Figure 4a, which we attribute to the relatively low (au(III))/(beta-ketoester (AEMA)) ratio employed. Notably, the size of the Au-NCs/SCNPs was found to be very stable over time, as illustrated in Table 1 and Figure 2a, showing the notorious stabilizing effect of the SCNPs against Au-NCs aggregation over time. Conversely, by using a (au(III))/(beta-ketoester (AEMA)) ratio of 1, the diameter of the resulting Au-NPs was found to grow with time, as illustrated in Figure 4b by the associated color changes. After 1 day, the DLS hydrodynamic diameter of the Au-NPs was found to be 67.1 nm.

Taken together, the above results demonstrate the efficient synthesis of stabilized Au-NCs with size < 5 nm within discrete poly(OEGMA-co-AEMA) SCNPs of ca. 11 nm in diameter. Key to the access to stabilized Au-NCs within SCNPs, instead of to larger Au-NPs that grow in size over time, is the control of the (Au(III))/(beta-ketoester (AEMA)) ratio employed during the synthesis.

### 3.2. Gold Nanoclusters within Single-Chain Nanoparticles (Au-NCs/SCNPs) as Catalytic Nanoreactors

We report herein the results of the use of Au-NCs within SCNPs as catalytic nanoreactors for the reduction of 4-nitrophenol, nitrobenzene, and 3-(4-nitrophenyl)-1,3-oxazolidin-2-one by borohydride (BH_4_^−^) in water at r.t.

#### 3.2.1. Reduction of 4-Nitrophenol to 4-Aminophenol Catalyzed by Au-NCs/SCNPs

**Figure 5:**
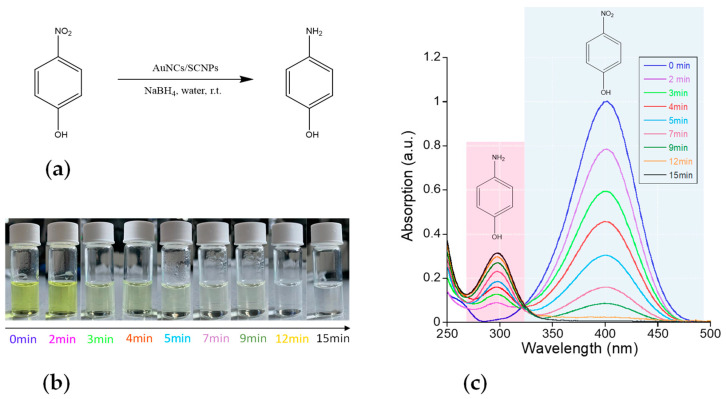
(**a**) Reduction of 4-nitrophenol to 4-aminophenol in water at r.t. by using the Au-NCs/SCNPs as highly efficient catalytic nanoreactors. (**b**) Color changes observed during the reduction of 4-nitrophenol (λ_max_ ≈ 400 nm) to 4-aminophenol (λ_max_ ≈ 300 nm) catalyzed by Au-NCs/SCNPs. (**c**) Kinetics of the reduction of 4-nitrophenol to 4-aminophenol catalyzed by Au-NCs/SCNPs as determined by UV-Vis spectroscopy.

Paracetamol—a popular analgesic and antipyretic agent used to treat fever and mild to moderate pain—can be typically synthesized from 4-nitrophenol as an intermediate via its reduction to 4-aminophenol and subsequent acetylation with acetic anhydride. 4-nitrophenol is highly soluble in water (11.6 mg/mL at 20 °C). We investigated the reduction of 4-nitrophenol (4-NP) to 4-aminophenol (4-AP) by BH_4_^−^ in water at r.t. by using the Au-NCs/SCNPs as highly efficient catalytic nanoreactors. This transformation has emerged as an important model reaction for assessing the catalytic activity of metallic nanoparticles in water. Section 2.3.3 details the experimental procedure that followed, which is depicted schematically in Figure 5a. The conversion of 4-aminophenol to 4-aminophenol was followed by UV-Vis spectrometry due to the different, well-separated absorption bands of the reactant and product, as illustrated in Figure 5b,c. Interestingly, under our reaction conditions, an isosbestic point located at ca. λ = 325 nm was observed in the UV-Vis spectra during the reduction of 4-aminophenol (λ_max_ ≈ 400 nm) to 4-aminophenol (λ_max_ ≈ 300 nm). This fact suggests the major involvement of a direct route mechanisms in the reduction of 4-nitrophenol to 4-aminophenol in water at r.t. with Au-NCs/SCNPs [27]. No reaction was observed in a model experiment lacking the Au-NCs/SCNPs catalyst.

**Figure 6:**
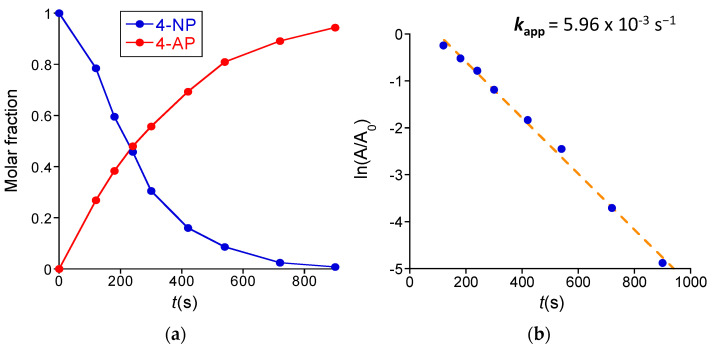
(**a**) Evolution of the concentration of reactant (blue) and product (red) over time (*t*) during the reduction of 4-nitrophenol (4-NP) to 4-aminophenol (4-AP) catalyzed by Au-NCs/SCNPs. (**b**) Determination of the apparent kinetic constant of the reduction of 4-NP to 4-AP catalyzed by Au-NCs/SCNPs (A/A_0_ is the 4-NP absorbance normalized to the absorbance at the start of the reaction).

Figure 6a shows the evolution of the concentration of the reactant and the product over time as estimated from data in Figure 5c, and the corresponding calibration curves are provided in the Supporting Information. A reaction yield of 95% was achieved in 15 min. of reaction time, where the amount of 4-nitrophenol was totally consumed (see Figure 5c). As illustrated in Figure 6b, the apparent kinetic constant (*k*_app_) of the reaction was estimated to be *k*_app_ = 5.96 × 10^−3^ s^−1^, which is a value between those reported by Yamamoto et al. for *N*,*N*-dimethylformamide-stabilized Au-NCs (*k*_app_ ≈ 3 × 10^−3^ s^−1^) and atomically monodisperse glutathione-stabilized Au-NCs (*k*_app_ ≈ 8 × 10^−3^ s^−1^) [28].

#### 3.2.2. Reduction of Nitrobenzene to Aniline Catalyzed by Au-NCs/SCNPs

**Figure 7:**
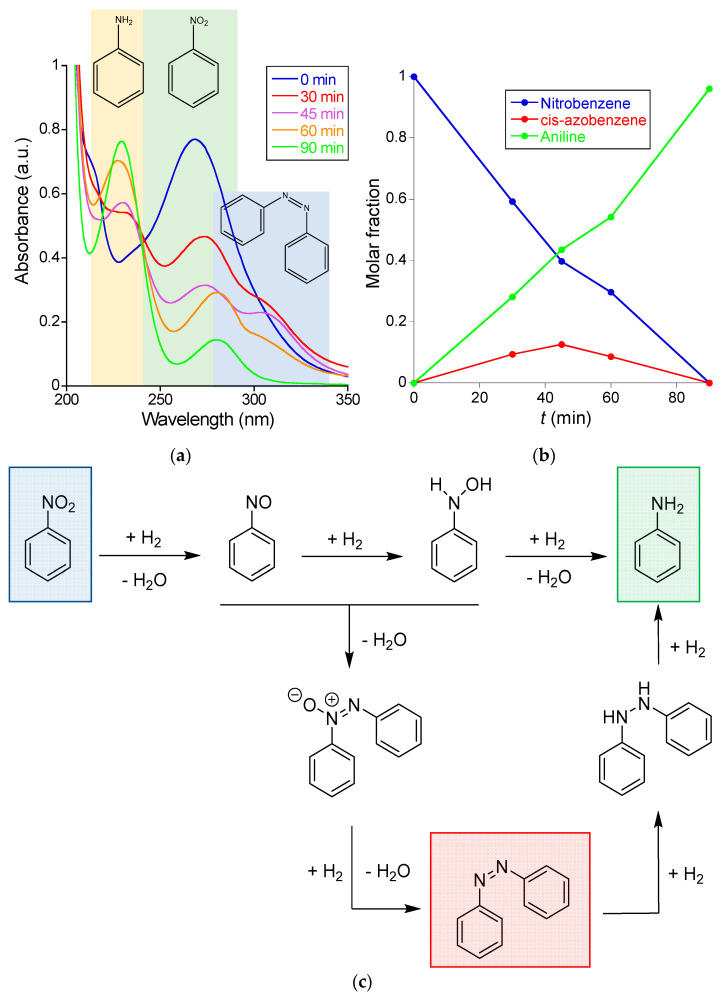
(**a**) Kinetics of the reduction of nitrobenzene to aniline catalyzed by Au-NCs/SCNPs as determined by UV-Vis spectroscopy. (**b**) Evolution of the concentration of nitrobenzene (blue), *cis*-azobenzene (red) and aniline (green) over time during the reduction of nitrobenzene to aniline catalyzed by Au-NCs/SCNPs. (**c**) Experimental data point to the involvement of both the direct route (**upper**) and the condensation route (**bottom**) mechanisms.

We also investigated the reduction in water at r.t. of nitrobenzene—which is sparingly water-soluble (1.9 mg/mL at 20 °C)—to aniline by BH_4_^−^ using the Au-NCs/SCNPs as catalytic nanoreactors. Figure 7a shows the UV-Vis spectra recorded at different reaction times during the nitrobenzene reduction reaction. Initially, the main UV-Vis absorption band of nitrobenzene is observed at λ_max_≈ 265 nm. The intensity of this band decreases with reaction time, and after 90 min., only the UV-Vis absorption peaks of aniline were clearly visible at λ_max_≈ 230 nm and 280 nm. However, during the reduction reaction process, the presence of a new UV-Vis absorption band centered at ca. λ_max_ ≈ 330 nm is clearly seen in Figure 7a. Based on the literature data [29], we attribute this new band to the generation of *cis*-azobenzene as an intermediate species that progressively transform to the aniline product (see Figure 7b). Consequently, in the case of the reduction in water at r.t. of nitrobenzene to aniline by BH_4_^−^ using the Au-NCs/SCNPs as catalytic nanoreactors, both the direct route and the condensation route mechanisms are involved [30]. Figure 7b provides the evolution of the concentration of nitrobenzene, *cis*-azobenzene, and aniline over time, as estimated from data in Figure 7a, and the corresponding calibration curves are provided in the Supporting Information. After 90 min. of reaction time, the reaction yield was 96% (see Figure 7b). In this case, the apparent kinetic constant (*k*_app_) of the reaction was *k*_app_ = 3.8 × 10^−4^ s^−1^ (see Supporting Information).

#### 3.2.3. Reduction of 3-(4-Nitrophenyl)-1,3-oxazolidin-2-one to 3-(4-Aminophenyl)-1,3-oxazolidin-2-one Catalyzed by Au-NCs/SCNPs

**Figure 8:**
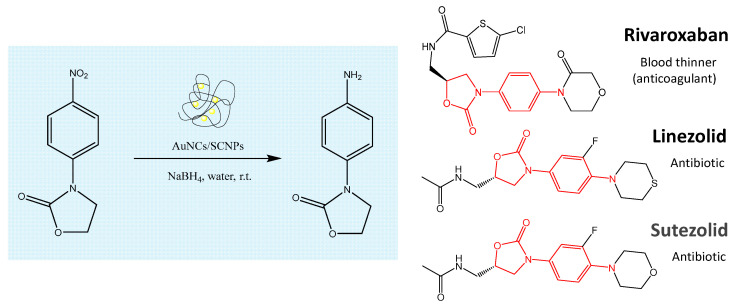
Illustration of the reduction of 3-(4-nitrophenyl)-1,3-oxazolidin-2-one to 3-(4-aminophenyl)-1,3-oxazolidin-2-one by BH_4_^−^ in water at r.t. catalyzed by Au-NCs/SCNPs. The aminophenyl–oxazolidinone fragment is a common motif contained in several drugs like Rivaroxaban, Linezolid, and Sutezolid.

Motivated by the high conversion observed during the Au-NCs/SCNPs-catalyzed reduction of both 4-nitrophenol and nitrobenzene, we additionally investigated the reduction of 3-(4-nitrophenyl)-1,3-oxazolidin-2-one to 3-(4-aminophenyl)-1,3-oxazolidin-2-one catalyzed by Au-NCs/SCNPs. In this sense, the aminophenyl–oxazolidinone fragment is a common motif contained in several drugs like Rivaroxaban, Sutezolid, and Linezolid (see Figure 8).

**Figure 9:**
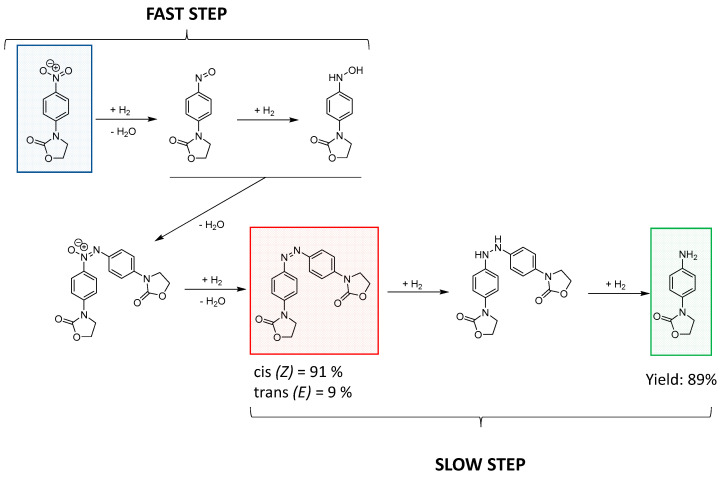
Reduction of 3-(4-nitrophenyl)-1,3-oxazolidin-2-one (in blue) to 3-(4-aminophenyl)-1,3-oxazolidin-2-one (in green) catalyzed by Au-NCs/SCNPs. The fast step corresponds to the formation of the diazene intermediate (in red), and the slow step to the generation of the aminophenyl-oxazolidinone product.

We found the Au-NCs/SCNPs-catalyzed reduction of 3-(4-nitrophenyl)-1,3-oxazolidin-2-one to proceed in two steps (fast and slow, respectively), as summarized in Figure 9.

**Figure 10:**
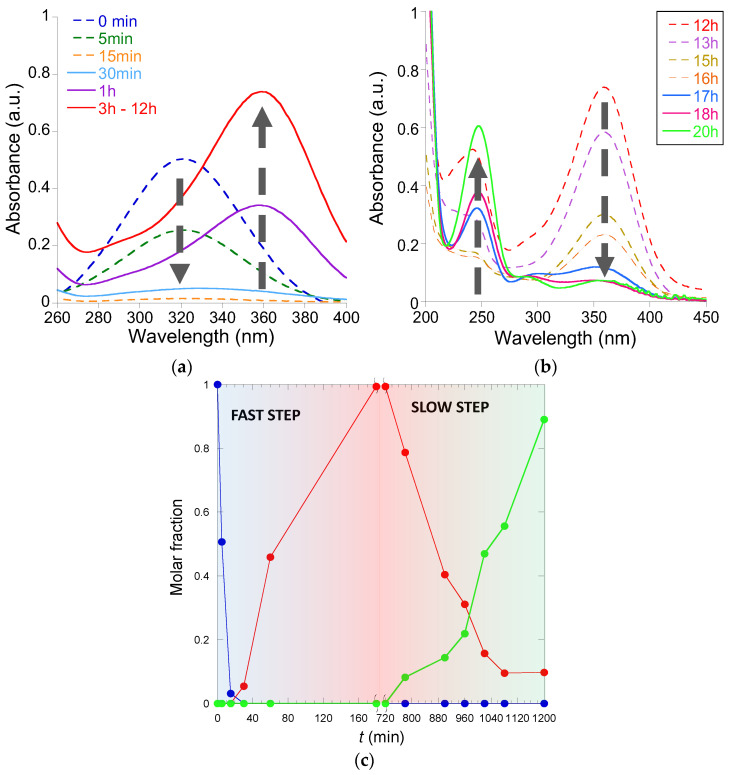
Kinetics of the reduction of 3-(4-nitrophenyl)-1,3-oxazolidin-2-one to 3-(4-aminophenyl)-1,3-oxazolidin-2-one catalyzed by Au-NCs/SCNPs as determined by UV-Vis spectroscopy corresponding to the fast step (**a**) and the slow step (**b**) of the reaction, as well as evolution of the concentration of 3-(4-nitrophenyl)-1,3-oxazolidin-2-one (blue color), (Z)-3,3′-(diazene-1,2-diylbis(4,1-phenylene))bis(oxazolidin-2-one) (red color), and 3-(4-aminophenyl)-1,3-oxazolidin-2-one (green color) over time (**c**).

Figure 10a shows the UV-Vis spectra recorded at different reaction times corresponding to the fast step. We observed the complete disappearance of the UV-Vis absorption peak of 3-(4-nitrophenyl)-1,3-oxazolidin-2-one (λ_max_ ≈ 320 nm) in 15 min. and then the appearance of a new UV-Vis absorption peak (λ_max_ ≈ 360 nm), which can be attributed to the generation of the diazene intermediate ((*Z*)-3,3′-(diazene-1,2-diylbis(4,1-phenylene))bis(oxazolidin-2-one)) in 3 h of reaction time. The intensity of this new band was stable after 3 h of reaction time, and it did not change until 9 h of additional reaction time. Then, during the slow step, a progressive disappearance of the UV-Vis peak of the diazene intermediate at λ_max_ ≈ 360 nm was found, as well as the concomitant appearance of a new UV-Vis absorption band at λ_max_ ≈ 250 nm corresponding to the 3-(4-aminophenyl)-1,3-oxazolidin-2-one product (see Figure 10b). Figure 10c illustrates the evolution of the concentration of 3-(4-nitrophenyl)-1,3-oxazolidin-2-one, (*Z*)-3,3′-(diazene-1,2-diylbis(4,1-phenylene))bis(oxazolidin-2-one), and 3-(4-aminophenyl)-1,3-oxazolidin-2-one over time, as estimated from data in Figure 10, and the corresponding calibration curves are provided in the Supporting Information. After 20 h of reaction time, the reaction yield was 89%.

## 4. Conclusions

We report the synthesis of gold nanoclusters (Au-NCs) with diameter < 5 nm (as revealed via TEM) within discrete single-chain polymeric nanoparticles (SCNPs) of poly(OEGMA-co-AEMA) with a diameter of around 11 nm (as revealed via DLS and TEM) and its further use as efficient catalytic nanoreactors for the reduction of model nitro-aromatic compounds in water at r.t. We show that the key to the access to stabilized Au-NCs within SCNPs, instead to larger Au-NPs that grow in size over time, is the control of the (au(III))/(beta-ketoester (AEMA)) ratio employed during the synthesis. As proof-of-concept of the catalytic activity of Au-NCs/SCNPs in water at r.t., we illustrate the fast reduction of nitrophenol to aminophenol, as well as the conversion of nitrobenzene to aniline in excellent yield. Finally, we show the efficient transformation in water of 3-(4-nitrophenyl)-1,3-oxazolidin-2-one to 3-(4-aminophenyl)-1,3-oxazolidin-2-one—a common motif contained in several drugs like Rivaroxaban, Sutezolid, and Linezolid—using the Au-NCs/SCNPs as catalytic nanoreactors. We hope this work will inspire the synthesis of other metal–NCs/SCNPs for advanced catalysis in aqueous media.

## Figures and Tables

**Table 1:** Evolution of average nanoparticle sizes during the synthesis of Au-NCs/SCNPs.

Material Type	Reaction Time, *t*	DLS Hydrodynamic Diameter, *D*_h_ (nm) ^2^
Poly(OEGMA-co-AEMA) ^1^	0 min.	11.0
Au-NCs/SCNPs	1 min.	11.0
Au-NCs/SCNPs	15 min.	10.7
Au-NCs/SCNPs	1 h	11.3
Au-NCs/SCNPs	1 day	11.2
Au-NCs/SCNPs	1 week	11.6

^1^ Self-assembled in water in the form of core–shell-like SCNPs. ^2^ Standard deviation ca. ± 0.3 nm.

## Data Availability

The data that support the findings of this study are available from the corresponding author upon reasonable request.

## Supplementary Materials

The following supporting information can be downloaded at https://www.mdpi.com/article/10.3390/polym16030378/s1: Figures S1–S5: Calibration curves for determination of the UV-Vis molar extinction coefficient of 4-nitrophenol, 4-aminophenol, nitrobenzene, cis-azobenzene and aniline; Figure S6: Apparent kinetic constant (kapp) of the reduction of nitrobenzene to aniline catalyzed by Au-NCs/SCNPs; Figures S7–S9: Calibration curves for determination of the UV-Vis molar extinction coefficient of 3-(4-nitrophenyl)-1,3-oxazolidin-2-one, (Z)-3,3′-(diazene-1,2-diylbis(4,1-phenylene))bis(oxazolidin-2-one) and 3-(4-aminophenyl)-1,3-oxazolidin-2-one; Figures S10–S13: 1H MNR spectra after isolation via preparative TCL of *cis*-azobenzene, aniline, (Z)-3,3′-(diazene-1,2-diylbis(4,1-phenylene))bis(oxazolidin-2-one) and 3-(4-aminophenyl)-1,3-oxazolidin-2-one.
