# Supplementary material for: Gold Nanoclusters Synthesized within Single-Chain Nanoparticles as Catalytic Nanoreactors in Water
Source: Polymers (Basel). 2024 Jan 30;16(3):378. doi: 10.3390/polym16030378 (PMC10857666; doi:10.3390/polym16030378)
Supplement: Supplementary file 1 [file polymers-16-00378-s001.zip › polymers-2813770-supplementary.pdf]

Supplementary Material

Gold Nanoclusters Synthesized within Single-Chain Nanoparticles as Catalytic Nanoreactors in Water

Jokin Pinacho-Olaciregui[1,2], Ester Verde-Sesto[1,3,] Daniel Taton[2] and José A. Pomposo[1,3,4,]*

[1]Centro de Física de Materiales (CSIC–UPV/EHU)–Materials Physics Center MPC, Pº Manuel Lardizabal 5, E-20018 Donostia, Spain.

[2]Laboratoire de Chimie des Polymères Organiques (LCPO), Université de Bordeaux INP–ENSCBP, 16 av. Pey Berland, 33607 Pessac cedex, France.

[3]IKERBASQUE–Basque Foundation for Science, Plaza Euskadi 5, E-48009 Bilbao, Spain.

[4]Departamento de Polímeros y Materiales Avanzados: Física, Química y Tecnología. University of the Basque Country (UPV/EHU), Pº Manuel Lardizabal 3, E-20800 Donostia, Spain.

* Corresponding author: josetxo.pomposo@ehu.eus

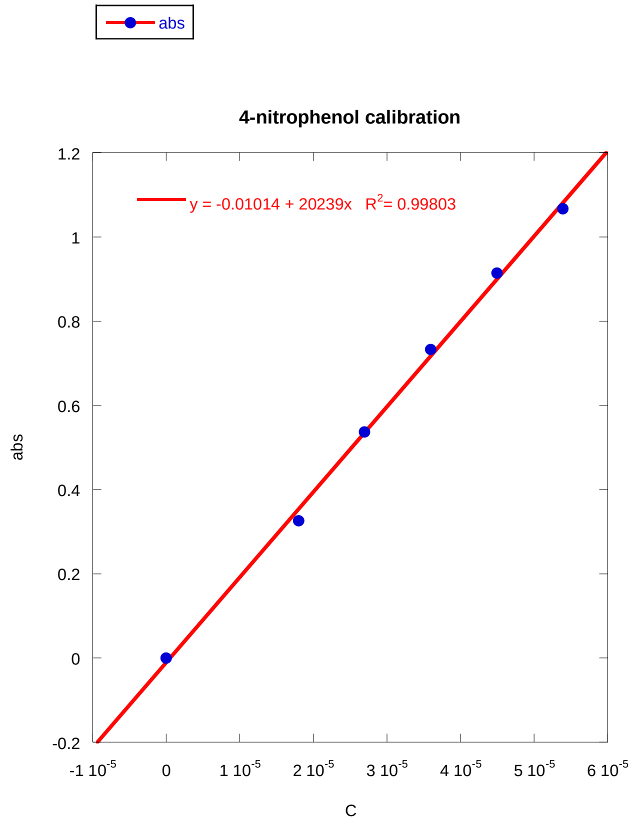

Figure S1. Calibration curve for determination of the UV-Vis molar extinction coefficient of 4-nitrophenol in water at r.t.: ε (λ_{max} = 400 nm) ≈ 20239 $M^{-1}cm^{-1}$. Literature value: ε (λ_{max} = 400 nm) ≈ 18900 $M^{-1}cm^{-1}$ (Coiffier A. *et al. J. Mater. Chem.* **2001**, *11*, 2039).

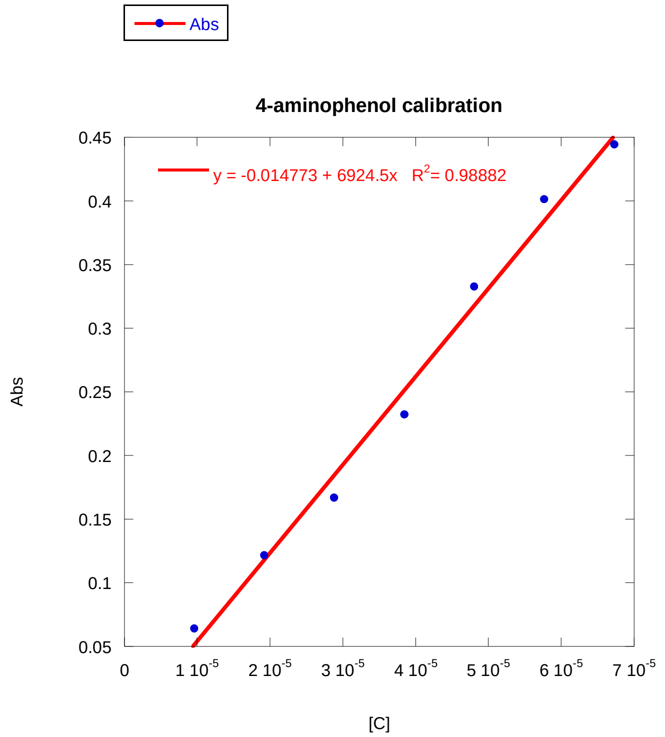

Figure S2. Calibration curve for determination of the UV-Vis molar extinction coefficient of 4-aminophenol in water at r.t.: ε (λ_{max} = 300 nm) ≈ 6925 $M^{-1}cm^{-1}$.

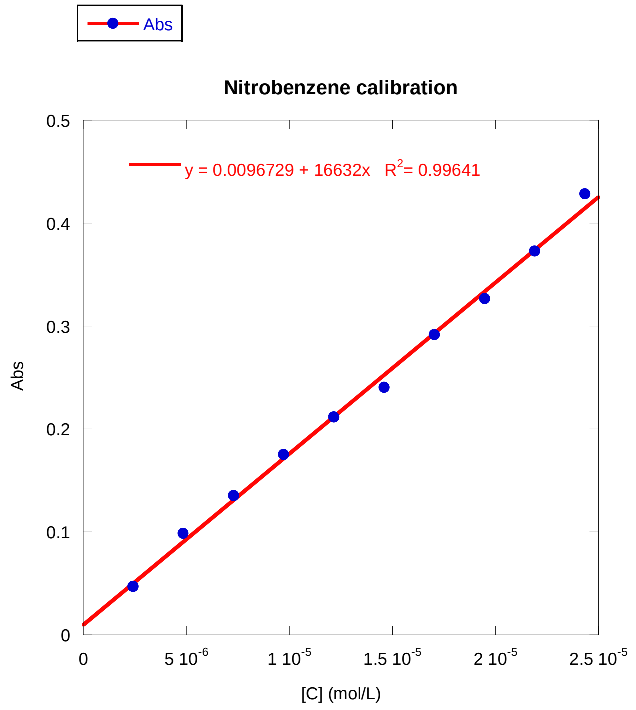

Figure S3. Calibration curve for determination of the UV-Vis molar extinction coefficient of nitrobenzene in water at r.t.: ε (λ_{max} = 265 nm) ≈ 16632 $M^{-1}cm^{-1}$.

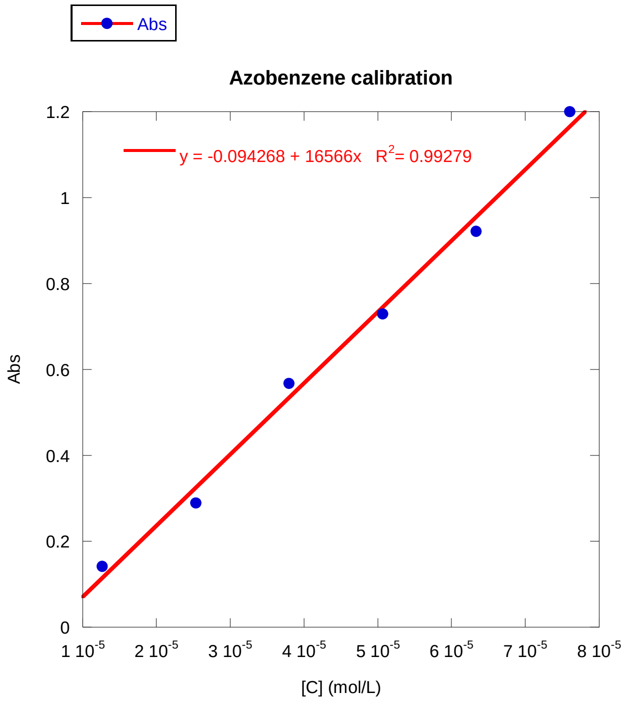

Figure S4. Calibration curve for determination of the UV-Vis molar extinction coefficient of azobenzene in water at r.t.: ε (λ_{max} = 230 nm) ≈ 16566 $M^{-1}cm^{-1}$.

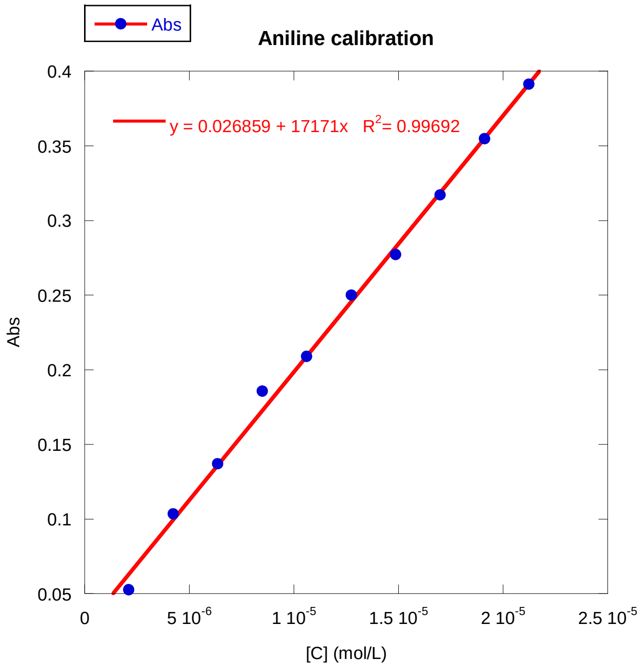

Figure S5. Calibration curve for determination of the UV-Vis molar extinction coefficient of aniline in water at r.t.: ε (λ_{max} = 230 nm) $\approx$ 20239 $M^{-1}cm^{-1}$.

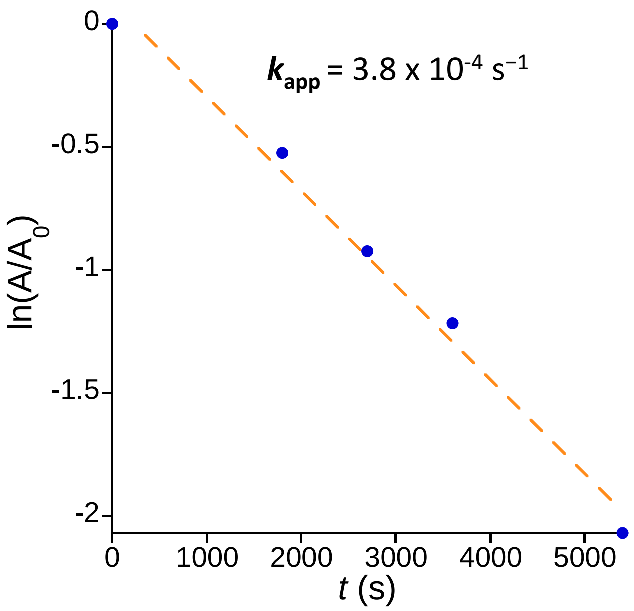

Figure S6. Apparent kinetic constant (k_{app}) of the reduction of nitrobenzene to aniline catalyzed by Au-NCs/SCNPs.

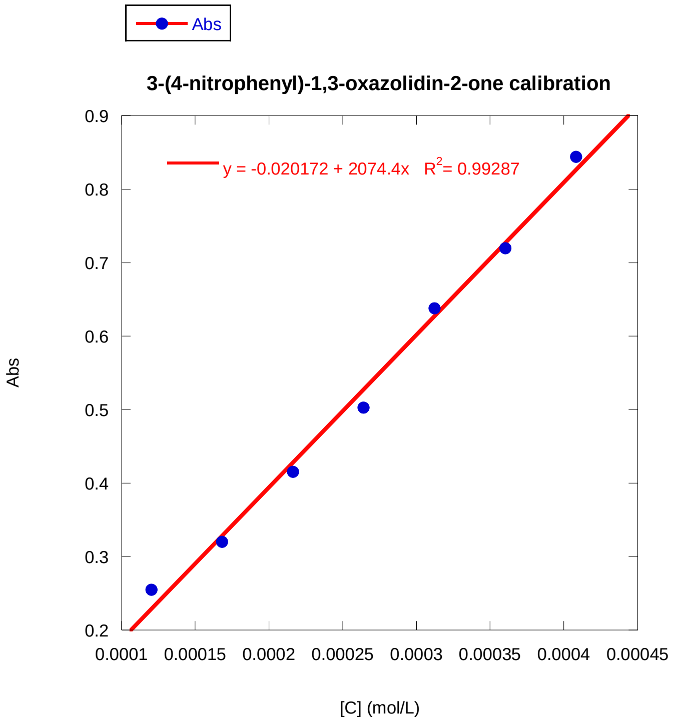

Figure S7. Calibration curve for determination of the UV-Vis molar extinction coefficient of 3-(4-nitrophenyl)-1,3-oxazolidin-2-one in water at r.t.: ε (λ_{max} = 320 nm) ≈ 2074 $M^{-1}cm^{-1}$.

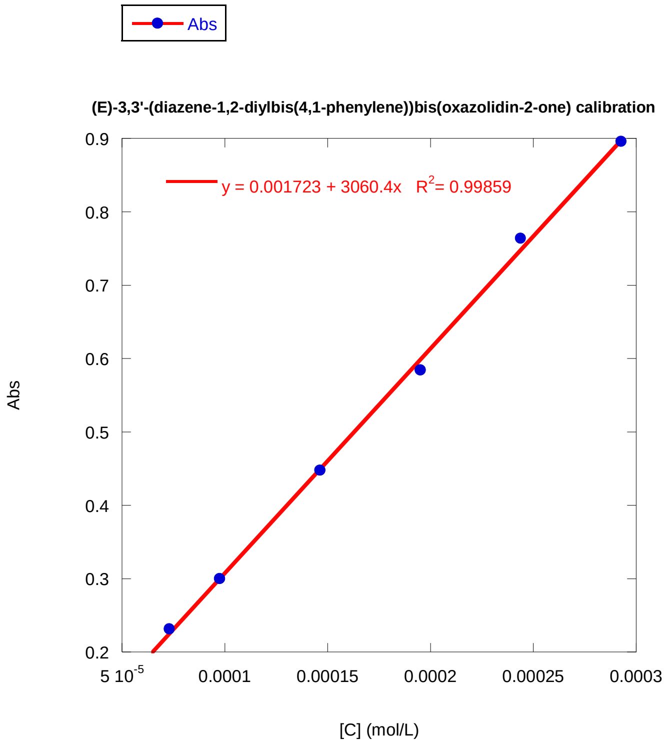

Figure S8. Calibration curve for determination of the UV-Vis molar extinction coefficient of (Z)-3,3'-(diazene-1,2-diylbis(4,1-phenylene))bis(oxazolidin-2-one) in water at r.t.: ε (λ_{max} = 360 nm) ≈ 3060 $M^{-1}cm^{-1}$.

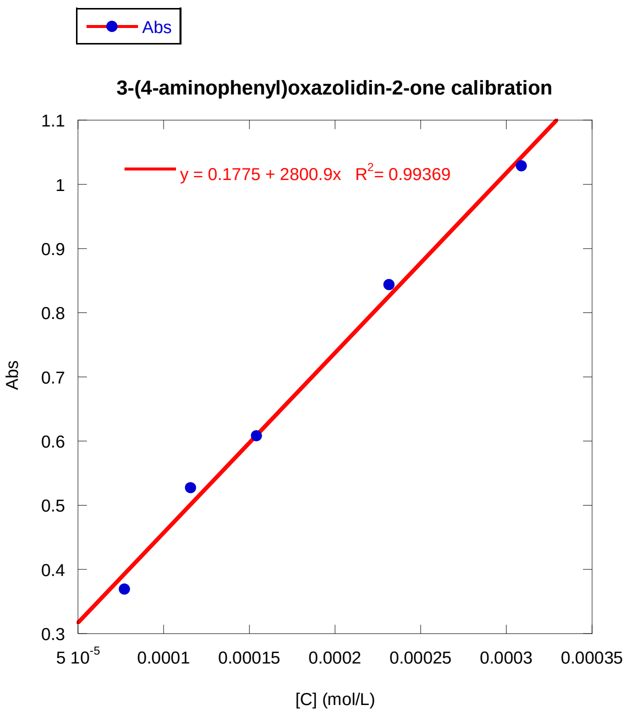

Figure S9. Calibration curve for determination of the UV-Vis molar extinction coefficient of 3-(4-aminophenyl)-1,3-oxazolidin-2-one in water at r.t.: ε (λ_{max} = 250 nm) ≈ 2801 $M^{-1}cm^{-1}$.

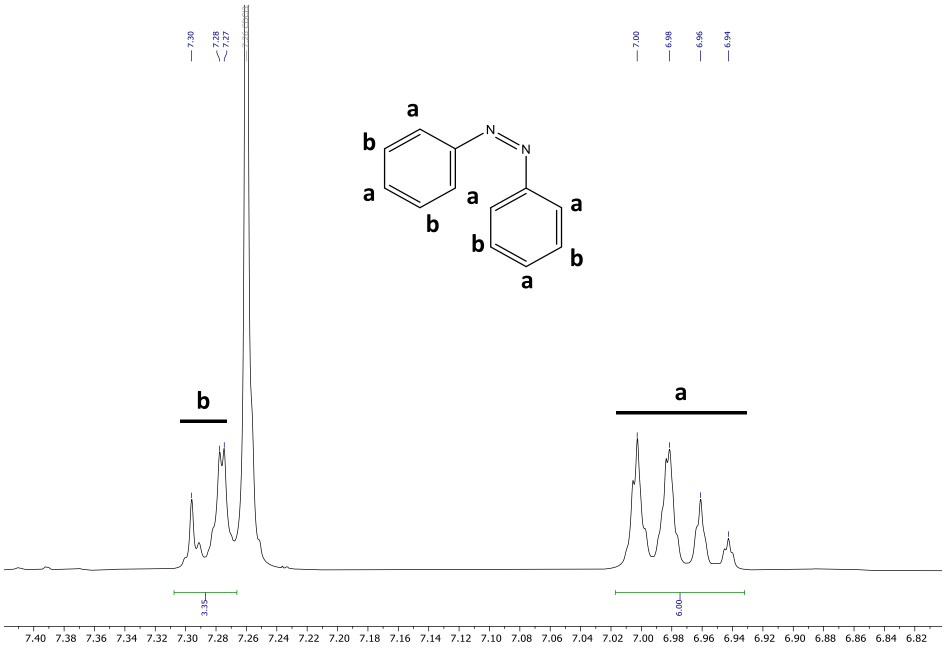

Figure S10. ^{1}H MNR spectrum after isolation *via* preparative TCL of the *cis*-azobenzene intermediate species.

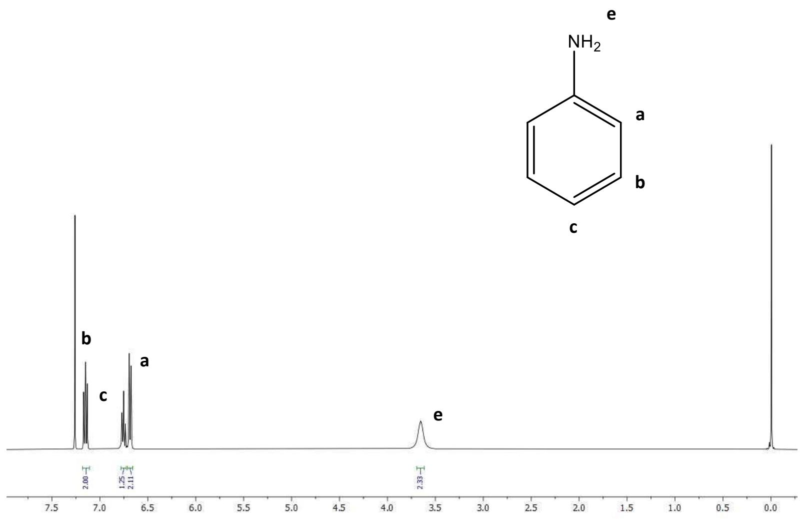

Figure S11. ^{1}H MNR spectrum after isolation *via* preparative TCL of the aniline product.

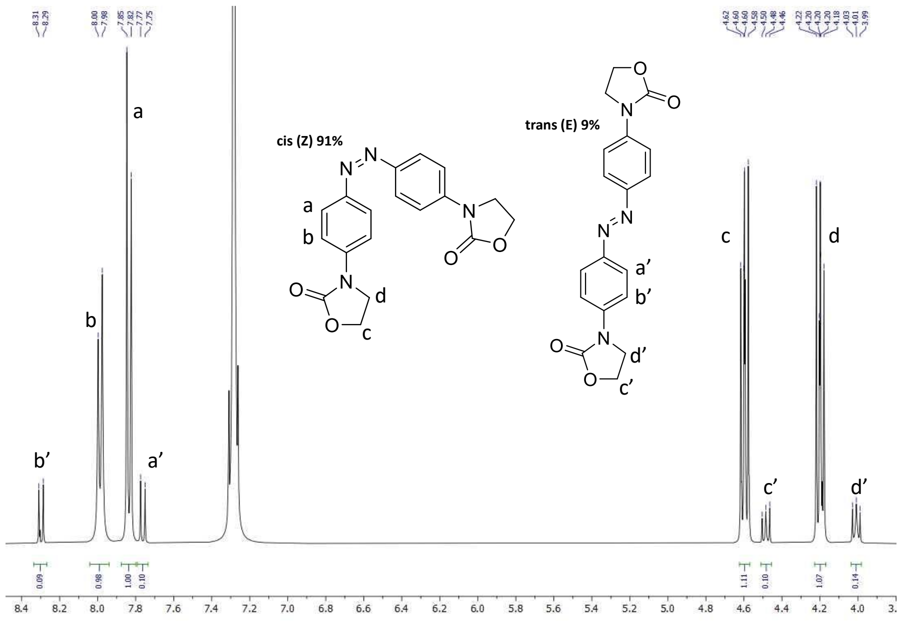

Figure S12. ^{1}H MNR spectrum after isolation *via* preparative TCL of the (Z)-3,3'-(diazene-1,2-diylbis(4,1-phenylene))bis(oxazolidin-2-one) intermediate species.

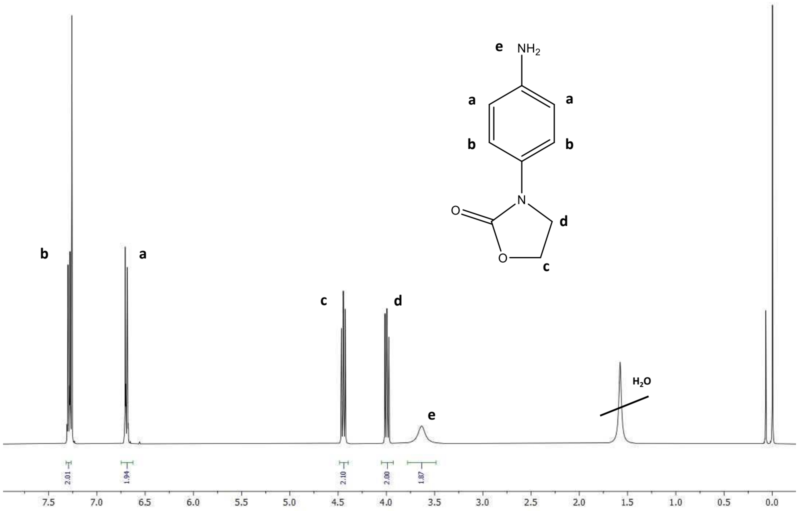

Figure S13. ^{1}H MNR spectrum after isolation *via* preparative TCL of the 3-(4-aminophenyl)-1,3-oxazolidin-2-one product.
